# Method for analyzing the clinical applicability of a terminology subset in primary health care: theoretical study^*^

**DOI:** 10.1590/1980-220X-REEUSP-2025-0098en

**Published:** 2025-12-12

**Authors:** Marcia Regina Cubas, Francine Dutra Mattei, Fernanda Broering Gomes Torres, Denilsen Carvalho Gomes

**Affiliations:** 1 Pontifícia Universidade Católica do Paraná, Programa de Pós-graduação em Tecnologia em Saúde, Curitiba, PR, Brazil.; 2 Pontifícia Universidade Católica do Paraná, Curso de Enfermagem, Curitiba, PR, Brazil.; 3 Secretaria Municipal da Saúde de São José dos Pinhais, Escola de Saúde Pública, São José dos Pinhais, PR, Brazil.

**Keywords:** Standardized Nursing Terminology, Methods, Primary Care Nursing

## Abstract

**Objective:**

To detail the stages of the method for analyzing the clinical applicability of terminological subsets in the context of Primary Health Care.

**Method:**

A theoretical- reflective study that presents three prerequisites and four stages of the method for analyzing the clinical applicability of terminological subsets, based on reflection on two operationalization experiences.

**Results:**

The prerequisites are related to the choice of the research setting, the selection of participants—clients and nurses, with an emphasis on inclusion criteria—and prior knowledge of the terminology and theoretical model. The steps of the method are: development of the data collection instrument, according to the steps of the Nursing Process and the categories of the theoretical model; systematic and participatory observation of the nursing consultation; evaluation of the return consultation at the health unit or at home; organization and analysis of data, in a descriptive manner and according to the theoretical model. In addition, a case study example can be developed. Final considerations: The method presented can be replicated in Primary Health Care settings, so that it can be critiqued, adjusted, and reorganized. It is understood that the method is a tool for analyzing praxis.

## INTRODUCTION

The International Council of Nurses (ICN) has proposed since 2008 that the use of the International Classification for Nursing Practice (ICNP^TM^) should be facilitated by terminological subsets targeted at specific clienteles or specialties or areas of nursing focus. The proposal is justified by the complexity and extent of ICNP^TM^, which makes it difficult to use in practice. Added to this problem is the incipient familiarity of nurses with classification systems represented by hierarchical ontological models. In the field of nursing, hierarchical ontological models enable the combination of primitive concepts to form pre-coordinated concepts. In the case of ICNP^TM^, nursing diagnoses, outcomes, and interventions are considered pre-coordinated concepts.

In order to develop subsets, the ICN presented in 2008 a 10-step guideline, followed by the publication of a six-step model related to the terminology life cycle. In 2015, the Brazilian method for developing subsets was released, with three prerequisites and four organizational steps^(1)^. Terminological subsets are relevant for representing nursing phenomena in reference terminologies, including the Systematized Nomenclature of Medicine - Clinical Terms (SNOMED-CT). Most of the pre-coordinated concepts in ICNP^TM^ 2019, which is represented by SNOMED-CT, originate from subsets approved by the ICN. A study comparing the representations of nursing care for people with COVID-19 in ICNP^TM^ 2019, SNOMED-CT, and the mapping of ICNP^TM^ with SNOMED-CT concluded that SNOMED-CT better covers the patient›s clinical situation^(2)^. This conclusion motivates the debate that ICNP^TM^ will improve the representation of its pre-coordinated concepts by benefiting from SNOMED-CT terms. For the improvement to be effective, research analyzing the applicability of subsets is necessary in order to identify the use of terms in healthcare practice, including those not found in ICNP^TM^ but found in SNOMED-CT.

The proposal for the Brazilian method in order to developing terminological subsets arose from a discussion about the problem of the lack of standardization and detail in the methodological approach in research on the topic^(1)^. The authors’ motivation was focused on providing a reproducible method whose results could be compared and reused in other national and international contexts.

Thus, despite the presence of the ICN guideline, considered the official method, two situations were visible in research that used the guideline. The first was that some of its steps were modified, excluded, or included. The second was that the reasons or justifications for such changes, exclusions, or inclusions were not explained. These situations discredited the scientific research that developed subsets and hindered the reproducibility of the method^(3)^.

Brazilian researchers are at the forefront of research on terminological subsets in various fields of knowledge and thematic areas of nursing. A literature review conducted between 2008 and 2017 identified 35 publications on the subject, of which 31 were linked to Brazilian postgraduate programs^(3)^. The article indicates the potential of these publications, including the presence of a model or theoretical framework that supported the subset. However, gaps are described, including the lack of specification or detail in the concept validation stage by experts and the incipient nature of clinical validation studies^(3)^. Thus, the question arises about the main purpose of a terminological subset: its application in clinical practice; therefore, clinical validation studies are considered essential for the consolidation of terminology^(4)^.

A scoping review on the clinical validation of ICNP^TM^ subsets, published in 2025, selected 15 studies, including articles, theses, and dissertations^(5)^. The main results described by the authors are: that Brazil is the largest producer of research on the topic, accounting for 14 of the 15 studies; 11 studies were developed in a hospital context and 10 used case studies as a method^(5)^. In the context of PHC, a study on the accuracy of clinical indicators in the phenomenon of chronic diseases was identified, and another study, aimed at the elderly, included direct observation of nursing consultations as a stage of the method^(5)^. Despite the careful application of the methods used in the selected studies, the authors conclude that there is a need for uniformity in the method, with the knowledge gap to be filled by this article.

It should be noted that, in the hospital environment the stages of the Nursing Process are carried out continuously during the hospitalization period, involving different nurses and teams. In Primary Health Care (PHC), the Nursing Process is operationalized at the time of the nursing consultation, performed by the nurse, which involves the health team in the stages that are legally incumbent upon them. The characteristics of practices in different contexts determine that the method of applicability analysis in the hospital is different from that in PHC. Thus, the differentiated detailing of the method is justified.

After the publication of the Brazilian method for developing terminological subsets in 2015, through a book chapter, and in 2017, through an article that compared the three available methods^(1)^, it has been used by several researchers. In the field of PHC, examples of subsets can be cited: for people with tuberculosis^(6)^ and for alcoholics^(7)^. In the hospital setting, examples of subsets include those for people with chronic kidney disease undergoing conservative treatment^(8)^ and for people with burns^(9)^. Although issues related to the details of the steps for developing subsets have been overcome, the limitation of the clinical validation step not being mandatory remains.

Ten years after the publication of the Brazilian method for developing subsets, it should be updated due to the migration from ICNP^TM^ to SNOMED-CT. In particular, this concerns the hierarchical ontological model of primitive concepts that supports cross-mapping between identified terms and terms in the reference classification. To date, there is no ICN position on how the subsets will be developed, which does not interfere with the fact that it is necessary to structure the analysis of the applicability of the subsets already developed in the Brazilian context.

The development of a method for clinical applicability analysis began through partnerships with developers of the subset for dying with dignity and the breastfeeding subset. Although the approaches were important and enlightening, the two experiences did not take place in PHC settings and used the term validation, which is not in line with the proposal for clinical applicability analysis in the PHC context. The first validated the subset for patients in palliative care in hospitals and hospices^(10)^. The second validated interventions in postpartum women in the immediate postpartum period and at the end of the period, through telephone calls^(11)^.

This corroborates the statement that the lack of clinical validation prevents the subset from accurately reflecting what it proposes to do^(3)^. In turn, studies that performed clinical validation of subsets reinforced two relevant limitations: a) traditional methods of clinical validation are primarily directed at one of the elements of the Nursing Process – diagnosis^(12,13)^ and, since the 2020s, nursing outcomes^(14)^; b) the studies are supported by a paradigm that is difficult to reproduce when dealing with a set of nursing diagnoses, outcomes, and interventions, specifically with regard to the number of participants and the sample size. This last limitation is not exclusive to ICNP^TM^. It was verified in the study that evaluated the content validity of the NANDA-I nursing diagnosis “Disrupted Family Processes” in incarcerated women, in which the authors indicate that the small sample size may generate biased values^(13)^. Thus, research on the clinical applicability of subsets should consider the sample issue as a barrier and seek alternatives to overcome it.

In turn, validation methods are generally related to studies that evaluate measurement instruments and are supported by psychometric analysis^(15)^. Thus, it is assumed that in studies with terminological subsets, the term validation may not achieve its full scope or, at best, it will be used in a restricted manner. Understanding that the main purpose is to analyze whether the subset has the potential to be used in the clinical practice of nurses, that is, in the operationalization of nursing consultations in PHC, we argue that the most appropriate term for the context is clinical applicability.

The objective of this article is to detail the steps of the method for analyzing the clinical applicability of terminological subsets in the context of Primary Health Care.

## METHOD

A theoretical-reflective study that presents the prerequisites and steps of a method for analyzing the clinical applicability of terminological subsets in the context of PHC.

A 2015 reflection on the validation of ICNP^TM(16)^ terminological subsets was used for the initial composition of the steps of the clinical applicability method in PHC. The authors of the reflection discussed validation models and suggested, in a preliminary and non-detailed manner, four steps that would be operationalized through clinical case studies. Of the suggested stages, the method described in this article used the development of the data collection instrument according to the theoretical model, with subsequent application of a pilot test; and the analysis of diagnoses, results, and interventions identified in practice^(16)^.

The method of clinical applicability of terminological subsets in PHC is an integral part of the reflective text defended by the first author in a competition for Associate Professor in the Department of Collective Health Nursing at the School of Nursing of the University of São Paulo in 2023. Its elaboration came from the inclusion of the method in a research project promoted by Public Call of the Universal Notice of the National Council for Technological Development/Ministry of Science and Technology/National Fund for Scientific and Technological Development (CNPq/MCTI/FNDCT) No. 18/2021.

The research resulting from the call for proposals was carried out in 2023 and 2024, in nursing consultations in primary health care in a municipality in the metropolitan region of Curitiba, Paraná, for adults with chronic pain. Additionally, the method was used to analyze the applicability of a child development subset, with children aged zero to three years, in the same municipality. To compose this article, descriptive texts of the method in the original projects were used, whose stages were detailed, justified, and adapted after application to the phenomena of chronic pain and child development.

The research that used the method was cleared by the Research Ethics Committee (CEP) of the Pontifical Catholic University of Paraná, under CAAE 56765322.1.0000.0020, on April 16, 2022, and CAAE 70495723.0.0000.0020, on August 11, 2023, and by the CEP of the São José dos Pinhais Health Secretariat, under CAAE 56765322.1.3002.9587, on May 2, 2023, and CAAE 70495723.0.3001.9587, on August 28, 2023.

## PREREQUISITES

Initially, it was noticed that some points precede the stages, being considered an indispensable requirement for the analysis of the applicability of the terminological subset in PHC. In this sense, three prerequisites are included: a) choice of study setting; b) selection of study participants; c) knowledge of the terminology, theoretical model, and operational definitions of the diagnoses and nursing outcomes of the terminological subset.

The prerequisites must be detailed and justified. In the first one—the choice of setting—the phenomenon to which the subset refers must be prevalent, or the clientele must be regular users of the study site. There is no doubt that if the phenomenon is not prevalent, there will be an obstacle to the selection of participants, and the identification of diagnoses, outcomes, and interventions may be hampered by the lack of defining attributes. It should be added that in research on the content validation of subset statements, using the Delphi technique, it was found that when the phenomena are in the nurse’s area of expertise, there is a tendency for the discussion to contribute more to the removal or addition of items^(8,17)^. Therefore, we defend the hypothesis that if the nursing practitioner recognizes the phenomenon in their routine, they will be able to identify the elements of the Nursing Process that can be represented by standardized terminology with greater clarity.

Regarding the second prerequisite - the selection of participants - it is important to consider appropriate criteria for nurses and users. Nurses who will be selected or invited to participate in the study must have clinical experience, that is, nursing consultations focused on the phenomenon or clientele must be part of their daily or weekly care routine. An inclusion criterion related to a minimum of two years as a nursing care nurse is suggested. This time frame was used in studies that applied the method described here. It was also used in a study that analyzed nursing diagnoses in home care^(18)^.

The clinical skill developed through the practice of nursing consultation is a topic steeped in contradictions. The length of training and work in PHC does not always guarantee clinical skills. This fact was observed in a study that analyzed the care practice of 463 nurses in Paraíba, in which the results describe that nursing consultation was reported as the main practice by 79.7% of nurses^(19)^. However, when the perception of effectiveness was analyzed, the result was 65%, with training and work time in PHC varying from one to 20 years^(19)^. Thus, it is suggested that the inclusion criteria for participating nurses should not be based solely on the length of training or work experience in PHC, but on the effective practice of nursing consultations with clients experiencing the phenomenon under study, including home visits. It is also strongly recommended that the researcher have experience in the operationalization of nursing consultations in PHC, otherwise it may represent a study bias due to a lack of identification with the Nursing Process.

Another suggestion to be evaluated when describing selection or inclusion criteria for nurses is academic training *lato* or *stricto sensu*. It is argued that for clinical applicability, having a postgraduate degree, although desirable, is not the central or main criterion for selection. In the proposal for instruments for selecting specialists for the development of terminological subsets, 13 criteria were established in the clinical applicability stage, distributed across five classification domains, with the domains of clinical experience and length of professional practice being assigned as relevant^(20)^.

In turn, the literature identifies that multiprofessional residency in family health has the potential to transform practices in services, despite the limitations related to the weaknesses of services and training paths^(21)^. It should be added that having a health residency was one of the criteria for including specialists in a study that identified ICNP^TM^ nursing diagnoses in people monitored in a home care program^(18)^. Thus, if in the study scenario it is possible to select nurses with training in family health residency, the criterion can be a qualifier for clinical applicability analysis, due to the nature of the training.

As for participating users who are seen by nursing consultations, it is necessary to carefully determine the inclusion criteria in order to adequately represent the phenomenon addressed. Thus, one of the suggested criteria is the user’s cognitive ability to understand simple information, as this can be a confounding factor in identifying subjective attributes or understanding nursing interventions. If it is impossible to include the criterion, an analysis of the confounding bias should be performed, indicating it as a limitation of the research. An alternative to minimize bias is to separate the results according to the cognitive characteristics of the participants.

Regarding the third prerequisite - knowledge of terminology, theoretical model, and operational definitions - a phase prior to data collection is suggested. In this phase, ICNP^TM^, the set of operational definitions, and the theoretical model will be presented. The way in which this moment will be operationalized is up to the researcher. As an example, in the analysis of the subset for people with chronic pain, nurses participated in three workshops with preparatory content, lasting eight hours each, and were participants in the research that validated the operational definitions^(22)^. This prerequisite is justified by the fact that ICNP® is not the hegemonic classification system in Brazil, therefore, the analysis of clinical applicability may be impaired by a lack of understanding of the components of the classification.

However, it is argued that the primitive terms of ICNP^TM^ represent everyday care situations, as shown by the results presented in the article that developed the subset for people with diabetic foot ulcers in PHC^(23)^. Internationally, Polish research that explored the use of ICNP^TM^ as part of professional development and learning, involving 772 nurses, concludes that despite the difficulties in implementing the classification in clinical practice, educational processes aid the development of skills and understanding for the use of ICNP^TM^ in the Nursing Process^(24)^. It is argued that the prior presentation also constitutes a strategy for continuing in-service education and that familiarizing nurses with standardized terminology can improve clinical reasoning, assist in the organized recording of phenomena, and enable the retrieval of structured information.

Regarding knowledge of the theoretical model, it is important to remember that terminological subsets are organized by the concepts of a theory or model, including medium-range theories (MRTs) developed specifically for them, such as the Interactive Breastfeeding Theory^(25)^ and the Theory of Care in the Context of Cardiovascular Risk^(26)^. In the context of PHC, a scoping review that mapped evidence on the use of theoretical frameworks by nurses identified 32 theories or models used in data collection, in the development of MRT or to support care^(27)^. The most widely used theory was Orem’s Self-Care Deficit Theory, followed by Roy’s Adaptation Theory, King’s Goal Attainment Theory, and Parse’s Theory^(27)^. The review discusses that PHC nurses apply the assumptions of major theories and, discreetly, MRT, emphasizing that the latter can meet care demands due to their ability to operate at the abstract level of major theories(27). It is considered that the approximation of PHC nurses with major theories, MRT or theoretical models used in the terminological subset enhances the understanding of the scope of their clinical applicability and supports the translation of knowledge.

Finally, we return to the statement about the criterion considered most relevant for choosing the participating nurse - clinical skill. The nurse’s experience with research involving ICNP^TM^, theories, or the Nursing Process is important for the development of a subset. However, the analysis of its application in practice is determined by the care nurse’s ability to recognize a phenomenon, plan and execute interventions, and analyze the expected or achieved results. If, during the selection process, a nurse with experience in research with ICNP^TM^ and/or the Nursing Process is identified, but with little clinical experience in PHC, it is up to the researcher to analyze the relevance of their participation and clearly justify their inclusion.

## STAGES

### Stage 1 - Development of the Data Collection Instrument

The form of data collection for clinical applicability analysis is systematic and participatory observation of the nursing consultation. The data collection instrument must be constructed according to the stages of the Nursing Process and organized according to the theoretical model that supported the subset. It should contain: a) items for initial assessment (sociodemographic data, medical history, anthropometric data, and physical examination), whose data have the potential to support the identification of nursing diagnoses, outcomes, and interventions; b) a list of nursing diagnoses, outcomes, and interventions for the subset; c) items for nursing progress. As this is a non-standardized instrument developed according to the scope of the terminological subset, a pilot test is necessary to verify the understanding of the questions and technical content and to adapt it to the routine of nursing consultations in the data collection scenario. The pilot test can be applied to nursing assistants with characteristics similar to those who will be selected for research, in order to verify the clarity and relevance of the items present in the instrument.

The data collection instrument should be completed by the researcher, but if other collectors are involved, training will be necessary for the use and guidance of systematic observation procedures. It is important to note that this is not an evaluation of how the nursing consultation is operationalized, but rather whether the terminological subset is applicable in healthcare practice. Thus, issues related to the form of the consultation (for example, whether the nurse performed the physical examination correctly) or resource items (for example, whether there are sufficient human or material resources) should not be included, nor should issues related to the management model (for example, whether nursing management supports the implementation of standardized terminologies). If it is necessary to contextualize the operationalization of the nursing consultation due to its relevance in the research scenario, the information should be included in the description of the study site.

At the risk of repeating ourselves, before data collection, nurses should have access to the terminological subset, structured according to the theoretical model. Any doubts need to be clarified, especially those related to classification, as it is not a hegemonic terminology in Brazilian nursing practice and teaching. Although there are specific experiences, such as the inclusion of ICNP^TM^ in PHC, combined with the International Classification of Primary Care (ICPC^®^), in the protocols of the state of Santa Catarina^(28)^. It is believed that unfamiliarity with ICNP^TM^, as well as lack of proximity to the content of the subset and the underlying theory, can be considered selection biases of the study participants. In other words, the terminological subset may not be applicable because nurses are unaware of the assumptions of the theory that underpinned it and are unfamiliar with its content.

### Stage 2 - Observation of the Nursing Appointment

Before describing this stage, we discuss the issue of the number of appointments to be observed. In a previous reflection on the topic, no quantitative suggestion was made^(16)^. In the two experiences of applicability analysis in hospital settings, the number was 20 people^(10,11)^. What changed in the research was the number of observations per person, given the characteristics of the study population, the phenomenon addressed, and the expected outcome.

In the hospital outpatient setting, an experience of clinical validation of a terminological subset for people with chronic kidney disease undergoing conservative treatment was iden- tified^(8)^. The study included in advance, 50 patients admitted and evaluated on admission, at 30 days, and at 60 days of hospitalization, whose data were used in the preparation of multiple case studies with subsequent analysis by specialist nurses in relation to the agreement with the diagnoses, results, and nursing interventions prepared by the researchers^(8)^. Although this is an excellent approach to the topic, given that the case studies are constructed with real data, there is still the limitation of including specialist nurses to analyze the terminological subset. Furthermore, the clinical applicability in consultations carried out by nursing assistants in the outpatient clinic is not analyzed, which is the gap addressed in this theoretical study.

In order to determine the number of participants in the analysis of the clinical applicability of the terminological subset in PHC, it is suggested to consider two questions: the number of consultations offered monthly by the nurse in the study setting and the forecast for care for clients experiencing the phenomenon in the same period. Based on this, initially, one can choose to observe all consultations performed in the month or for a longer period until at least 20 consultations are reached. In the two experiences of operationalizing the method, no sample calculation was established, and the collection of the 20 initial and return consultations took an average of three months, with weekly visits to the health unit.

The nursing appointment will be conducted by the health unit nurse and observed by the researcher, without interference. The nursing consultation user should be informed about the researcher’s role in this process and about the research, as they are also a participant in the research. Thus, the free and informed consent form will be offered to the nurse and the person being consulted, respecting the ethical requirements of research involving human beings.

The diagnoses, expected results, and nursing interventions will be identified by the researcher and the nurse individually and without exchanging information. The nurse will make the record in the medical record, according to the routine of the health unit. It is important to note that the fact that the service does not have a specific space in the medical record for recording the elements that make up the terminological subset does not prevent the research from being carried out, as it is expected that the recording of the stages of the Nursing Process will be carried out, in accordance with the specific legislation of the profession.

After the consultation is over, the researcher and the nurse will compare the diagnoses, expected outcomes, and interventions selected and indicate the similarity, agreement, or disagreement between the elements (diagnoses, outcomes, and interventions) observed by the researcher, identified by the nurse, and recorded by the nurse.

In addition to identifying similarity, agreement, or disagreement, the comparison aims to check the observation made and the records kept. The following should be grouped for further analysis: a) elements observed by the researcher and not identified by the nurse; b) elements identified by the nurse and not observed by the researcher; c) elements identified by the nurse and not recorded; d) elements recorded by the nurse and not observed by the researcher; e) elements observed by the researcher, identified, and recorded by the nurse.

### Step 3 - Evaluation at the Follow-up Appointment

One or more follow-up appointments for subsequent evaluation will be scheduled at the end of the first appointment in order to comply with the nursing evolution step of the Nursing Process. The suggested time frame is up to 30 days, depending on the phenomenon, clientele, or theoretical model. The need for more than one follow-up appointment depends on the nursing diagnosis identified and the timing of the proposed intervention.

The follow-up appointment can take place at the health unit or at home and should be conducted, as a priority, by the nurse who conducted the initial appointment. If it is not possible for them to conduct the appointment, it may be conducted by the researcher who collected the data. The follow-up consultation will identify: the result achieved or sensitive to the proposed intervention, adherence to the interventions, and the identification, if relevant, of a new diagnosis.

It should be noted that the follow-up nursing appointment guides professional practice, allowing for the systematic monitoring of health phenomena and conditions and enabling the assessment of the person/family’s adherence to the proposed interventions. This topic has been addressed in recent research, such as a study that identified self-management of exercise and medication adherence in at-risk patients in PHC^(29)^, which allowed the steps of the Nursing Process to be carried out in their entirety.

### Step 4 - Data Organization and Analysis

It is suggested that after each appointment, the data be entered into a spreadsheet immediately and checked by another researcher independently. The collected data should be organized or categorized according to the theoretical model and described in absolute and relative frequency of the elements of nursing practice.

It should be noted that data related to nursing interventions, due to the number of statements, can be presented together, but always respecting the theoretical model and the nursing diagnosis to which it refers. It is important to analyze which intervention was related to the nursing outcome identified in the subsequent consultation - this will provide support for analyzing outcomes sensitive to nursing action.

Additionally, the data identified in the initial assessment and return consultation will be used to compose example case studies with a description of all stages of the Nursing Process related to the two nursing consultations. The development of example case studies will assist in the analysis of the adequacy of diagnoses, outcomes, and interventions that were considered applicable and will allow their use in continuing health education.

The elements applicable to practice are those identified by the researcher or nurse, regardless of whether they are recorded in the medical record. At this stage of knowledge production on clinical applicability analysis, it is not considered relevant to establish a minimum frequency for an element to be considered applicable, but it will be a necessary step in order to produce research with more general results.

To compose the analysis, we return to the theoretical model that supported the subset and suggest that the contradictions between the model, the principles and guidelines of the Unified Health System, and the care model that is hegemonic in the study scenario be evaluated. After the analysis, it is possible to adapt and update the subset so that it truly represents the phenomenon, clientele, or specialty to which it refers.

Finally, it is argued that the reference terminology model of ISO 18.104 represents detailed clinical model structures with great potential to contribute to the analysis of applicability. This situation was observed in the research that identified attributes of the focus and judgment axes and the physical and psychological domains for the evaluation of perinatal care nursing^(30)^, whose model was used to categorize attributes and domains. As a future study, the method described here should use the content of ISO 18.104 to improve data collection and analysis of results.

**Figure 1:**
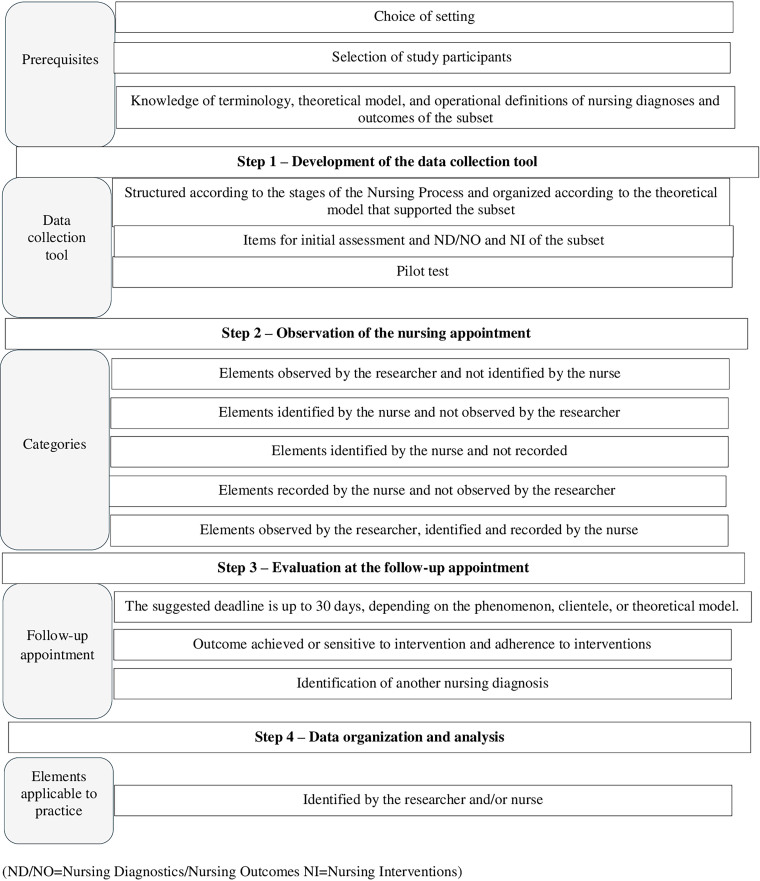
Summary table of the steps in the method for analyzing the clinical applicability of terminological subsets in PHC. Curitiba, 2025.

A summary table of the methodological path for assessing the clinical applicability of a terminological subset in PHC is shown in Figure 1.

## LIMITATIONS OF THE METHOD

As an innovative and little-explored method, it has limitations. In terms of prerequisites, there may be difficulties in: a) selecting nurses with clinical skills; b) operationalizing continuing education spaces with the theme of ICNP^TM^ and theoretical models. With regard to the observation of consultations, one limitation to be considered is the fact that the researcher must also have experience with nursing consultations. These scenarios are difficult to converge. Furthermore, the method lacks a debate related to the number of consultations observed depending on the prevalence of the phenomenon or the number of nursing consultations performed in the analyzed context. Another limitation refers to the absence of statistical criteria for sample size. It is believed that, with the progressive use of the method, the limitations will be overcome. Finally, the incipient nature of international studies on the topic limits comparisons between countries.

## FINAL CONSIDERATIONS

The method described consists of three prerequisites and four steps. It can be replicated in PHC settings so that it can be critiqued, adjusted, and reorganized. It is believed that subsets submitted to clinical applicability assessment have the potential to provide accuracy to the elements that compose them and to represent the needs of the people, families, or communities for whom the subsets are intended. It is considered that the implementation of a classification system to represent nursing practices in PHC is no guarantee that the nursing team will establish a care or work process that adheres to the worldview of collective health, nor to the theoretical model that underpins the subset. For this, it is necessary to incorporate changes in the hegemonic model and discussions/appropriations of conceptual theoretical assumptions, both from specific nursing knowledge derived from its own theories and from the field of collective health. However, it is understood that the method is a tool for analyzing praxis.

## Data Availability

The entire dataset supporting the results of this study was published in the article itself.
