# Supplementary material for: Method for analyzing the clinical applicability of a terminology subset in primary health care: theoretical study
Source: Rev Esc Enferm USP. 2025 Dec 12;59:e20250098. doi: 10.1590/1980-220X-REEUSP-2025-0098en (PMC12715867; doi:10.1590/1980-220X-REEUSP-2025-0098en)
Supplement: Translation

# Translation: Método de análise de aplicabilidade clínica de subconjunto terminológico na atenção primária em saúde: estudo teórico^*^

**DOI:** 10.1590/1980-220X-REEUSP-2025-0098pt

**Authors:** Marcia Regina Cubas, Francine Dutra Mattei, Fernanda Broering Gomes Torres, Denilsen Carvalho Gomes

**Affiliations:** 1 Pontifícia Universidade Católica do Paraná, Programa de Pós-graduação em Tecnologia em Saúde, Curitiba, PR, Brasil.; 2 Pontifícia Universidade Católica do Paraná, Curso de Enfermagem, Curitiba, PR, Brasil.; 3 Secretaria Municipal da Saúde de São José dos Pinhais, Escola de Saúde Pública, São José dos Pinhais, PR, Brasil.

**Keywords:** Terminologia Padronizada em Enfermagem, Métodos, Enfermagem de Atenção Primária

## Abstract

**Objetivo:**

Detalhar as etapas do método para análise de aplicabilidade clínica de subconjuntos terminológicos no contexto da Atenção Primária à Saúde.

**Método:**

Estudo teórico-reflexivo que apresenta três pré-requisitos e quatro etapas do método para análise de aplicabilidade clínica de subconjuntos terminológicos, a partir da reflexão sobre duas experiências de operacionalização.

**Resultados:**

Os pré-requisitos estão relacionados à escolha do cenário da pesquisa, à seleção dos participantes – clientela e enfermeiros, com ênfase nos critérios de inclusão, e ao conhecimento prévio da terminologia e do modelo teórico. As etapas do método são: elaboração do instrumento de coleta de dados, de acordo com etapas do Processo de Enfermagem e das categorias do modelo teórico; observação, sistemática e participante, da consulta de enfermagem; avaliação da consulta de retorno na unidade de saúde ou no domicílio; organização e análise dos dados, de modo descritivo e de acordo com o modelo teórico. De forma complementar, pode ser elaborado estudo de caso exemplo. Considerações finais: O método apresentado poderá ser replicado aos cenários da Atenção Primária à Saúde, de modo a ser criticado, ajustado e reorganizado. Compreende-se que o método é um instrumento para analisar a práxis.

## INTRODUÇÃO

Desde 2008, o *International Council of Nurses* (ICN) propõe que o uso da Classificação Internacional para a Prática de Enfermagem (CIPE®) seja facilitado por subconjuntos terminológicos direcionados para determinada clientela ou especialidade ou foco de atenção da enfermagem. A proposta é justificada pela complexidade e extensão da CIPE®, que a torna de difícil utilização na prática. Soma-se à problemática, a incipiente familiaridade dos enfermeiros com sistemas classificatórios representados por modelos hierárquicos ontológicos. No domínio da Enfermagem, os modelos hierárquicos ontológicos possibilitam a combinação entre os conceitos primitivos para composição de conceitos pré-coordenados. No caso da CIPE®, os diagnósticos, os resultados e as intervenções de enfermagem são considerados conceitos pré-coordenados.

Para elaboração de subconjuntos o ICN apresentou, em 2008, um *guideline* constituído de 10 passos, posteriormente foi publicado um modelo de seis passos relacionados ao ciclo de vida da terminologia e, em 2015, foi divulgado o método brasileiro para desenvolvimento de subconjuntos com três pré-requisitos e quatro etapas organizadoras^(1)^. Os subconjuntos terminológicos são relevantes para a representação dos fenômenos de enfermagem em terminologias de referência, dentre a quais, a *Systematized Nomenclature of Medicine - Clinical Terms* (SNOMED-CT). A maioria dos conceitos pré-coordenados da CIPE® 2019, que está representada pela SNOMED-CT, tem origem nos subconjuntos aprovados pelo ICN. Estudo que comparou as representações dos cuidados de enfermagem às pessoas com COVID-19 na CIPE® 2019, na SNOMED-CT e no mapeamento da CIPE® com a SNOMED-CT, concluiu que a SNOMED-CT abrange melhor a situação clínica do paciente^(2)^. Tal conclusão motiva o debate de que a CIPE® melhorará a representação de seus conceitos pré-coordenados ao se beneficiar dos termos da SNOMED-CT. Para que a melhora seja efetiva, pesquisas de análise da aplicabilidade de subconjuntos são necessárias, de modo a identificar o uso de termos na prática assistencial, incluindo os não constantes na CIPE®, mas constantes na SNOMED-CT.

A proposta do método brasileiro para desenvolvimento de subconjuntos terminológicos nasceu de uma discussão acerca da problemática da falta de padronização e detalhamento do percurso metodológico nas pesquisas sobre o tema^(1)^. A motivação das autoras foi centrada no fornecimento de um método capaz de ser reproduzido, cujos resultados gerados fossem passíveis de comparação e reutilização em outros contextos nacionais e internacionais.

Destarte a presença do *guideline* do ICN, considerado como o método oficial, duas situações eram visíveis em pesquisas que utilizaram o *guideline*. A primeira, que algumas de suas etapas eram modificadas, excluídas ou incluídas. A segunda, que os motivos ou justificativas para tais mudanças, exclusões ou inclusões não eram explicitados. Tais situações ofereciam descrédito científico às pesquisas que desenvolviam subconjuntos e dificultavam a reprodutibilidade do método^(3)^.

Pesquisadores brasileiros protagonizam a produção de pesquisas sobre subconjuntos terminológicos em vários domínios de conhecimento e áreas temáticas da Enfermagem. Uma revisão de literatura conduzida entre os anos de 2008 e 2017, identificou 35 produções sobre o assunto, destas, 31 vinculadas a programas brasileiros de pós-graduação^(3)^. No artigo são indicados os potenciais das produções, dentre eles, a presença de modelo ou referencial teórico que sustentou o subconjunto. Porém, lacunas são descritas, dentre elas, a falta de especificação ou detalhamento da etapa de validação dos conceitos por especialistas e a incipiência de estudos de validação clínica^(3)^. Assim, levanta-se a questão acerca da principal finalidade de um subconjunto terminológico: sua aplicação na prática clínica; portanto, estudos de validação clínica são considerados essenciais para consolidação da terminologia^(4)^.

Uma revisão de escopo sobre validação clínica de subconjuntos da CIPE®, publicada em 2025, selecionou 15 estudos, dentre artigos, teses e dissertações^(5)^. Os principais resultados descritos pelos autores são: que o Brasil é o maior produtor de pesquisas sobre o tema, sendo responsável por 14 dos 15 estudos; 11 estudos foram desenvolvidos no contexto hospitalar e 10 utilizaram estudo de caso como método^(5)^. No contexto da APS, identificou-se um estudo de acurácia de indicadores clínicos, no fenômeno das doenças crônicas, e outro estudo, direcionado à pessoa idosa, a observação direta da consulta de enfermagem foi incluída como etapa do método^(5)^. Apesar da aplicação criteriosa dos métodos utilizados nas pesquisas selecionadas, os autores concluem que há necessidade de uniformidade no método^(5)^, sendo a lacuna de conhecimento a ser suprida pelo presente artigo.

Destaca-se que no ambiente hospitalar as etapas do Processo de Enfermagem são desenvolvidas de forma contínua durante o período de hospitalização, incluindo diferentes enfermeiros e equipes. Já na Atenção Primária à Saúde (APS), o Processo de Enfermagem é operacionalizado no momento da consulta de enfermagem, realizada pelo enfermeiro, que envolve a equipe de saúde nas etapas que lhe cabem legalmente. As características das práticas nos distintos contextos determinam que o método de análise de aplicabilidade no hospital seja distinto ao da APS. Deste modo, justifica-se o detalhamento diferenciado do método.

Após a publicação do método brasileiro para desenvolvimento de subconjuntos terminológicos, em 2015, por meio de capítulo de livro e, em 2017, pelo artigo que comparou os três métodos disponíveis^(1)^, ele tem sido utilizado por vários pesquisadores. No domínio da APS é possível citar exemplos dos subconjuntos: para a pessoa com tuberculose^(6)^ e para a pessoa alcoolista^(7)^. Na área hospitalar são exemplos os subconjuntos: para pessoas com doença renal crônica em tratamento conser- vador^(8)^ e para pessoas com queimaduras^(9)^. Embora as questões relacionadas ao detalhamento de etapas para o desenvolvimento de subconjuntos tenham sido superadas, persiste o limite da não obrigatoriedade da etapa de validação clínica.

Após 10 anos da publicação do método brasileiro para desenvolvimento de subconjuntos, ele deverá ser atualizado em decorrência da migração da CIPE® para SNOMED-CT. Em especial, no que se refere ao modelo ontológico hierárquico dos conceitos primitivos que sustenta o mapeamento cruzado entre os termos identificados e os termos da classificação de referência. Até o momento, não há um posicionamento do ICN sobre como os subconjuntos serão desenvolvidos, o que não interfere no fato de que é necessário estruturar a análise de aplicabilidade dos subconjuntos já elaborados no contexto brasileiro.

A elaboração de um método para análise de aplicabilidade clínica iniciou-se por meio de parcerias com desenvolvedores do subconjunto para morrer com dignidade e do subconjunto de amamentação. Embora as aproximações tenham sido importantes e esclarecedoras, as duas experiências não aconteceram em espaços da APS e usaram o termo validação, não se aproximando da proposta da análise de aplicabilidade clínica no contexto da APS. A primeira, validou o subconjunto para pacientes em cuidados paliativos em hospital e *hospice* ^(10)^. A segunda, validou as intervenções em puérperas no pós-parto imediato e ao final do período, por meio de ligação telefônica^(11)^.

Corrobora-se a afirmação de que a inexistência de validação clínica impede que o subconjunto possa refletir exatamente aquil a que se propõe^(3)^. Por sua vez, as pesquisas que realizaram validação clínica de subconjuntos reforçaram duas limitações relevantes: a) os métodos tradicionais de validação clínica se direcionam, prioritariamente, para um dos elementos do Processo de Enfermagem – o diagnóstico^(12,13)^ e, a partir da década de 2020, ao resultado de enfermagem^(14)^; b) as pesquisas estão suportadas por um paradigma de difícil reprodução em se tratando de um conjunto de diagnóstico, resultado e intervenção de enfermagem, em específico no que se refere ao número de participantes e da amostra. Esta última limitação não é exclusiva da CIPE®. Ela foi verificada no estudo que avaliou a validade de conteúdo do diagnóstico de enfermagem “Processos Familiares Interrompidos” da NANDA-I, em mulheres privadas de liberdade, no qual os autores indicam que o pequeno número da amostra pode gerar valor tendencioso^(13)^. Assim, as pesquisas de aplicabilidade clínica de subconjunto devem considerar a questão da amostra como uma barreira e buscar alternativas para superá-la.

Por sua vez, métodos de validação estão relacionados, de maneira geral, às pesquisas que avaliam instrumentos de medida e possuem suporte na análise psicométrica^(15)^. Deste modo, defende-se o pressuposto que nas pesquisas com subconjuntos terminológicos, o termo validação pode não atingir seu alcance ou, na melhor das hipóteses, ele será utilizado de forma restrita. Entendendo que a finalidade principal é analisar se o subconjunto tem potencial para ser usado na prática clínica de enfermeiros, ou seja, na operacionalização da consulta de enfermagem na APS, defende-se que o termo mais adequado ao contexto é aplicabilidade clínica.

O objetivo deste artigo é detalhar as etapas do método para análise de aplicabilidade clínica de subconjuntos terminológicos, no contexto da Atenção Primária à Saúde.

## MÉTODO

Estudo teórico-reflexivo, que apresenta os pré-requisitos e as etapas de um método para análise de aplicabilidade clínica de subconjuntos terminológicos, no contexto da APS.

Uma reflexão, de 2015, sobre validação dos subconjuntos terminológicos da CIPE®^(16)^ foi utilizada para composição inicial das etapas do método de aplicabilidade clínica na APS. As autoras da reflexão debateram modelos de validação e sugeriram, de forma preliminar e não detalhada, quatro etapas que seriam operacionalizadas por meio de estudos de casos clínicos. Das etapas sugeridas, o método descrito no presente artigo utilizou a elaboração do instrumento de levantamento de dados de acordo com o modelo teórico, com posterior aplicação de teste piloto; e a análise dos diagnósticos, resultados e intervenções identificadas na prática^(16)^.

O método de aplicabilidade clínica de subconjuntos terminológicos na APS é parte integrante do texto reflexivo defendido pela primeira autora em concurso de Livre Docente no Departamento de Enfermagem em Saúde Coletiva (ENS) da Escola de Enfermagem da Universidade de São Paulo (EEUSP), em 2023. Sua elaboração adveio da inclusão do método em um projeto de pesquisa fomentado por Chamada Pública do Edital Universal do Conselho Nacional de Desenvolvimento Tecnológico/Ministério da Ciência e Tecnologia/Fundo Nacional de Desenvolvimento Científico e Tecnológico (CNPq/ MCTI/FNDCT) nº 18/2021.

A pesquisa decorrente do edital foi operacionalizada nos anos de 2023 e 2024, em consultas de enfermagem na APS de um município da região metropolitana de Curitiba - Paraná, para adultos com dor crônica. Adicionalmente, o método foi usado para analisar aplicabilidade de subconjunto de desenvolvimento infantil, com crianças de zero a três anos de idade, no mesmo município. Para compor o presente artigo, foram utilizados os textos descritivos do método nos projetos de origem, cujas etapas foram detalhadas, justificadas e adequadas após a aplicação nos fenômenos da dor crônica e desenvolvimento infantil.

As pesquisas que utilizaram o método foram aprovadas pelo Comitê de Ética em Pesquisa (CEP) da Pontifícia Universidade Católica do Paraná, sob CAAE 56765322.1.0000.0020, em 16 de abril de 2022, e CAAE 70495723.0.0000.0020, em 11 de agosto de 2023, e pelo CEP da Secretaria de Saúde de São José dos Pinhais, sob CAAE 56765322.1.3002.9587, em 2 de maio de 2023, e CAAE 70495723.0.3001.9587, em 28 de agosto de 2023.

## PRÉ-REQUISITOS

Inicialmente, percebeu-se que alguns pontos antecedem as etapas, sendo considerados exigência indispensável à análise de aplicabilidade de subconjunto terminológico na APS. Neste sentido, são incluídos três pré-requisitos: a) escolha do cenário de estudo; b) seleção dos participantes do estudo; c) conhecimento da terminologia, do modelo teórico e das definições operacionais dos diagnósticos e resultados de enfermagem do subconjunto terminológico.

É preciso detalhar e justificar os pré-requisitos. No primeiro – a escolha do cenário, o fenômeno a que se refere o subconjunto deve ser prevalente ou a clientela deve ser usuária habitual do local de estudo. Não há dúvidas que se o fenômeno não for prevalente, haverá obstáculo para seleção de participantes e a identificação dos diagnósticos, resultados e intervenções pode ser prejudicada pela falta de atributos definidores. Acrescenta-se a justificativa de que em pesquisas sobre validação de conteúdo dos enunciados de subconjunto, por meio de técnica Delphi, verificou-se que quando os fenômenos são da área de domínio do enfermeiro há tendência da discussão com maior contribuição para retirada ou acréscimo de itens^(8,17)^. Portanto, defende-se a hipótese de que se o enfermeiro assistencial reconhece o fenômeno em sua rotina, ele será capaz de identificar os elementos do Processo de Enfermagem passíveis de representação por terminologia padronizada, com maior clareza.

Em relação ao segundo pré-requisito – a seleção dos participantes, é importante considerar critérios adequados para os enfermeiros e para os usuários. Os enfermeiros que serão selecionados ou convidados para participar do estudo devem, obrigatoriamente, ter experiência clínica, ou seja, a consulta de enfermagem direcionada para o fenômeno ou clientela deve ser uma rotina assistencial diária ou semanal. Sugere-se um critério de inclusão relacionado ao tempo como enfermeiro assistencial de no mínimo dois anos. Esta temporalidade foi usada nas pesquisas que aplicaram o método aqui descrito. Também foi usada na pesquisa que analisou diagnósticos de enfermagem na assistência domiciliar^(18)^.

A habilidade clínica desenvolvida pelo exercício da consulta de enfermagem é um tema imerso em contradições. Nem sempre o tempo de formação e trabalho na APS garante habilidade clínica. Fato que foi observado em um estudo que analisou a prática assistencial de 463 enfermeiros Paraibanos, no qual os resultados descrevem que a consulta de enfermagem foi relatada como prática principal por 79,7% dos enfermeiros^(19)^. Entretanto, quando se analisou a percepção de resolutividade, o resultado foi de 65%, sendo que o tempo de formação e de trabalho na APS variou de um a 20 anos^(19)^. Deste modo, sugere-se que o critério de inclusão dos enfermeiros participantes não seja direcionado apenas para o tempo de formação ou o tempo de trabalho na APS, mas para o efetivo exercício da consulta de enfermagem à clientela que vivencia o fenômeno pesquisado, incluindo as consultas realizadas no domicílio. Também se recomenda, fortemente, que o pesquisador tenha experiência na operacionalização da consulta de enfermagem na APS, caso contrário pode representar um viés de estudo, pela falta de identidade com o Processo de Enfermagem.

Outra sugestão a ser avaliada ao descrever critérios de seleção ou inclusão dos enfermeiros é a formação acadêmica *lato* ou *stricto sensu*. Defende-se que para aplicabilidade clínica, ter uma pós-graduação, embora seja desejável, não é o critério central ou principal para escolha. Na proposta de instrumentos para seleção de especialistas para desenvolvimento de subconjuntos terminológicos foram estabelecidos 13 critérios na etapa de aplicabilidade clínica distribuídos em cinco domínios classificadores, sendo os domínios da experiência clínica e do tempo de prática profissional atribuídos como relevantes^(20)^.

Por sua vez, é identificado na literatura que a residência multiprofissional em saúde da família tem potencial para transformar práticas nos serviços, apesar dos limites relacionados às fragilidades dos serviços e percursos formativos^(21)^. Acrescenta-se que possuir residência em saúde foi um dos critérios de inclusão de especialistas em um estudo que identificou diagnósticos de enfermagem da CIPE® em pessoas acompanhadas em programa de assistência domiciliar^(18)^. Dessa forma, se no cenário de estudo houver possibilidade de selecionar enfermeiros com a formação de residência em saúde da família, o critério pode ser um qualificador para análise de aplicabilidade clínica, pela característica da formação.

Quanto aos usuários participantes, atendidos pela consulta de enfermagem, é preciso determinar com parcimônia os critérios de inclusão de modo a representar adequadamente o fenômeno abordado. Assim, um dos critérios sugeridos é a capacidade cognitiva do usuário para compreender informações simples, pois ela pode ser um elemento de confusão para identificação de atributos subjetivos ou para compreensão das intervenções de enfermagem. Na impossibilidade de incluir o critério, uma análise do viés de confusão deve ser feita, indicando-o como limite da pesquisa. Uma alternativa para minimizar viés é separar os resultados, de acordo com as características cognitivas dos participantes.

Em relação ao terceiro pré-requisito – o conhecimento da terminologia, do modelo teórico e das definições operacionais, sugere-se uma fase anterior à coleta dos dados. Nela, a CIPE®, o conjunto das definições operacionais e o modelo teórico serão apresentados. A forma como este momento será operacionalizado é de escolha do pesquisador. Como exemplo, na análise do subconjunto para pessoas com dor crônica, as enfermeiras participaram de três oficinas com conteúdo preparatório, com duração de oito horas cada, e foram participantes da pesquisa que validou as definições operacionais^(22)^. Este pré-requisito se justifica pelo fato de a CIPE® não ser o sistema classificatório hegemônico no Brasil, portanto, a análise de aplicabilidade clínica pode ser prejudicada pelo não entendimento dos componentes da classificação.

Entretanto, discute-se que os termos primitivos da CIPE® representam situações do cotidiano da assistência, a exemplo dos resultados apresentados no artigo que elaborou o subconjunto para pessoas com úlcera de pé diabético na APS^(23)^. No âmbito internacional, pesquisa realizada na Polônia, que explorou o uso da CIPE® como parte do desenvolvimento e aprendizado profissional, envolvendo 772 enfermeiros, apresenta em sua conclusão de que apesar das dificuldades para implementação da classificação na prática clínica, os processos educacionais auxiliam o desenvolvimento de habilidades e a compreensão para uso da CIPE® no Processo de Enfermagem^(24)^. Defende-se que o momento prévio de apresentação também se configura em estratégia de educação permanente em serviço e a aproximação dos enfermeiros com terminologias padronizadas pode melhorar o raciocínio clínico, auxiliar o registro do fenômeno de forma organizada e possibilitar a recuperação de informações estruturadas.

Quanto ao conhecimento do modelo teórico, é importante relembrar que os subconjuntos terminológicos são organizados pelos conceitos de uma teoria ou modelo, incluindo as teorias de médio alcance (TMA) elaboradas especificamente para eles, a exemplo da Teoria Interativa de Amamentação^(25)^ e da Teoria do Cuidado no Contexto de Risco Cardiovascular^(26)^. No contexto da APS, uma revisão de escopo que mapeou evidências sobre a utilização de referenciais teóricos por enfermeiros, identificou 32 teorias ou modelos empregados na coleta de dados, na elaboração de TMA ou para subsidiar o cuidado^(27)^. A teoria mais utilizada foi a teoria do Déficit de Autocuidado de Orem, seguida pelas teorias de Adaptação de Roy, de Alcance de Metas de King e a Teoria de Parse^(27)^. Na revisão discute-se que enfermeiros da APS aplicam os pressupostos das grandes teorias e, de forma discreta, as TMA, salientando que estas últimas podem atender demandas de cuidado pela capacidade de operar no nível abstrato das grandes teorias^(27)^. Considera-se que a aproximação dos enfermeiros da APS com grandes teorias, TMA ou modelos teóricos utilizados no subconjunto terminológico potencializa a compreensão do alcance de sua aplicabilidade clínica e sustenta a translação de conhecimento.

Por fim, retoma-se a afirmação sobre o critério considerado de maior relevância para escolha do enfermeiro participante – a habilidade clínica. A experiência do enfermeiro com as pesquisas envolvendo a CIPE®, as teorias ou o Processo de Enfermagem são importantes para o desenvolvimento de um subconjunto. Entretanto, a análise de sua aplicação na prática é determinada pela capacidade que o enfermeiro assistencial possui para reconhecer um fenômeno, planejar e executar intervenções e analisar os resultados esperados ou atingidos. Caso no processo de seleção se identifique enfermeiro com experiência em pesquisa com a CIPE® e/ou Processo de Enfermagem, mas com pouca experiência clínica na APS, cabe ao pesquisador analisar a pertinência de sua participação e deixar clara a justificativa para incluí-lo.

## ETAPAS

### Etapa 1 - Elaboração do Instrumento de Coleta de Dados

A forma de coleta de dados para análise de aplicabilidade clínica é a observação sistemática e participante da consulta de enfermagem. O instrumento de coleta de dados deve ser construído conforme as etapas do Processo de Enfermagem e organizado de acordo com o modelo teórico que sustentou o subconjunto. Ele deverá conter: a) itens para avaliação inicial (dados sociodemográficos, anamnese, dados antropométricos e exame físico), cujos dados possuem potencial para subsidiar a identificação de diagnósticos, resultados e intervenções de enfermagem; b) lista de diagnósticos, resultados e intervenções de enfermagem do subconjunto; c) itens para a evolução de enfermagem. Por se tratar de instrumento não padronizado e elaborado de acordo com o escopo do subconjunto terminológico, é necessário um teste-piloto para verificação de compreensão das questões e do conteúdo técnico e para adequá-lo à rotina da consulta de enfermagem no cenário de coleta de dados. O teste-piloto pode ser aplicado aos enfermeiros assistenciais com características semelhantes aos que serão selecionados para pesquisa, a fim de verificar a clareza e pertinência dos itens presentes no instrumento.

O instrumento de coleta de dados deve ser preenchido pela pesquisadora, mas, caso envolver outros coletadores será necessário treinamento para uso e balizamento dos procedimentos da observação sistemática. É importante afirmar que não se trata de uma avaliação sobre a forma como a consulta de enfermagem é operacionalizada, mas sim, se o subconjunto terminológico é aplicável na prática assistencial. Desse modo, questões relacionadas à forma da consulta (como exemplo, se o enfermeiro realizou exame físico corretamente) ou itens de recursos (como exemplo, se há recursos humanos ou materiais suficientes) não devem ser incluídas, tampouco, as questões relacionadas ao modelo de gestão (como exemplo, se a chefia de enfermagem apoia a implantação de terminologias padronizadas). Sugere-se que se for necessário contextualizar a operacionalização da consulta de enfermagem pela relevância dela no cenário de pesquisa, as informações devem estar na descrição do local de estudo.

Correndo o risco de repetição, antes da coleta de dados, os enfermeiros devem ter acesso ao subconjunto terminológico, estruturado de acordo com o modelo teórico. As dúvidas precisam ser esclarecidas, principalmente, as relacionadas à classificação, pois ela não é uma terminologia hegemônica na prática e no ensino da enfermagem brasileira. Embora existam experiências pontuais, como é o caso da inclusão da CIPE® na APS, conjugada o com a Classificação Internacional de Atenção Primária (CIAP®), nos protocolos do estado de Santa Catarina^(28)^. Acredita-se que o desconhecimento da CIPE®, bem como a não proximidade com o conteúdo do subconjunto e da teoria de base podem ser considerados vieses de seleção dos participantes do estudo. Ou seja, o subconjunto terminológico pode não ser aplicável porque os enfermeiros não sabem os pressupostos da teoria que o sustentou e desconhecem seu conteúdo.

### Etapa 2 - Observação da Consulta de Enfermagem

Antes de descrever essa etapa, discute-se a problemática do número de consultas a serem observadas. Em reflexão anterior sobre o tema não é sugerido quantitativo^(16)^. Nas duas experiências de análise de aplicabilidade em espaços hospitalares, o número foi de 20 pessoas^(10,11)^. O que modificou nas pesquisas foi o número de observações por pessoa, dadas as características da população do estudo, do fenômeno abordado e do desfecho esperado.

No espaço ambulatorial hospitalar, identificou-se experiência de validação clínica de subconjunto terminológico para pessoas com doença renal crônica em tratamento conservador^(8)^. Na pesquisa foram incluídos, antecipadamente, 50 pacientes internados e avaliados na admissão, em 30 dias e em 60 dias de internação, cujos dados foram usados na elaboração de estudos de casos múltiplos com posterior análise por enfermeiros especialistas em relação à concordância com os diagnósticos, resultados e intervenções de enfermagem elaborados pelos pesquisadores^(8)^. Embora seja uma excelente aproximação com o tema, pelo fato de os estudos de caso serem construídos com dados reais, persiste a limitação da inclusão de enfermeiros especialistas para analisar o subconjunto terminológico. Também, não se analisa a aplicabilidade clínica nas consultas praticadas por enfermeiros assistenciais no ambulatório, que é a lacuna contemplada no presente estudo teórico.

Para determinar o número de participantes na análise de aplicabilidade clínica de subconjunto terminológico na APS sugere-se ponderar dois quesitos: o número de consultas oferecidas mensalmente pelo enfermeiro do cenário de estudo e a previsão de atendimento à clientela que vivencia o fenômeno, no mesmo período. A partir disso, em um primeiro momento, pode-se optar pela observação de todas as consultas realizadas no mês ou por um tempo maior até que se atinjam, no mínimo, as 20 consultas. Nas duas experiências de operacionalização do método não foi estabelecido cálculo de amostra e a coleta das 20 consultas, iniciais e de retorno, tiveram tempo de médio de três meses, com idas semanais à unidade de saúde.

A consulta de enfermagem será conduzida pelo enfermeiro da unidade de saúde e observada pelo pesquisador, sem interferência. O usuário da consulta de enfermagem deve ser orientado sobre o papel do pesquisador neste processo e sobre a pesquisa, pois ele também é participante da pesquisa. Dessa forma, o termo de consentimento livre e esclarecido será oferecido para o enfermeiro e para pessoa consultada, sendo respeitados os requisitos éticos de pesquisa que envolve seres humanos.

Os diagnósticos, resultados esperados e intervenções de enfermagem serão identificados pelo pesquisador e pelo enfermeiro, de forma individual e sem troca de informações. O enfermeiro fará o registro em prontuário, conforme a rotina da unidade de saúde. Importante salientar que o fato de o serviço não dispor de espaço específico no prontuário para registro dos elementos que compõe o subconjunto terminológico não impede que a pesquisa seja realizada, pois espera-se que o registro das etapas do Processo de Enfermagem seja realizado, conforme legislação específica da profissão.

Após a finalização da consulta, o pesquisador e o enfermeiro farão a comparação entre os diagnósticos, resultados esperados e intervenções selecionados e indicarão a similaridade, concordância ou discordância entre os elementos (diagnósticos, resultados e intervenções) observados pelo pesquisador, identificados pelo enfermeiro e registrados pelo enfermeiro.

Além de identificar similaridade, concordância ou discordância, a comparação tem a finalidade de checar a observação realizada e os registros realizados. Devem ser agrupados para posterior análise: a) elementos observados pelo pesquisador e não identificados pelo enfermeiro; b) elementos identificados pelo enfermeiro e não observados pelo pesquisador; c) elementos identificados pelo enfermeiro e não registrados; d) elementos registrados pelo enfermeiro e não observados pelo pesquisador; e) elementos observados pelo pesquisador, identificados e registrados pelo enfermeiro.

### Etapa 3 - Avaliação na Consulta de Retorno

Uma ou mais consultas de retorno para avaliação subsequente serão agendadas ao final da primeira consulta, de modo a cumprir a etapa de evolução de enfermagem do Processo de Enfermagem. O prazo sugerido é de até 30 dias, a depender do fenômeno, clientela ou do modelo teórico. A necessidade de mais de uma consulta de retorno depende do diagnóstico de enfermagem identificado e da temporalidade da intervenção proposta.

A consulta de retorno pode acontecer na unidade de saúde ou no domicílio e deverá ser feita, prioritariamente, pelo enfermeiro que realizou a consulta inicial. Na impossibilidade de ele conduzir a consulta, ela poderá ser realizada pelo pesquisador que coletou os dados. Na consulta de retorno serão identificados: o resultado alcançado ou sensível à intervenção proposta, a adesão às intervenções e a identificação, caso pertinente, de novo diagnóstico.

Ressalta-se que a consulta de enfermagem de retorno orienta a prática profissional permitindo o acompanhamento sistemático dos fenômenos e condições de saúde e possibilita a avaliação da adesão da pessoa/família às intervenções propostas. Este tema tem sido abordado em pesquisas recentes, a exemplo de estudo que identificou o autogerenciamento da prática de exercícios e adesão medicamentosa, em pacientes de risco na APS^(29)^, que permitiu a execução das etapas do Processo de Enfermagem em sua completude.

### Etapa 4 - Organização e AnáLise dos Dados

Sugere-se que após cada consulta os dados sejam digitados em planilha eletrônica, imediatamente, e checados por outro pesquisador de forma autônoma. Os dados coletados devem ser organizados ou categorizados conforme o modelo teórico e descritos em frequência absoluta e relativa dos elementos da prática de enfermagem.

Salienta-se que os dados relacionados às intervenções de enfermagem, em virtude da quantidade de enunciados, podem ser apresentados em conjunto, mas sempre respeitando o modelo teórico e o diagnóstico de enfermagem a que ele se refere. Importante analisar qual intervenção teve relação com o resultado de enfermagem identificado na consulta subsequente – isso dará suporte para analisar resultados sensíveis à ação de enfermagem.

Adicionalmente, os dados identificados na avaliação inicial e na consulta de retorno serão utilizados para compor estudos de caso exemplo com a descrição de todas as etapas do Processo de Enfermagem relacionadas às duas consultas de enfermagem. A elaboração de estudo de caso exemplo auxiliará na análise de adequação dos diagnósticos, resultados e intervenções que foram considerados aplicáveise permitirá o uso em momentos de educação permanente em saúde.

Considera-se que os elementos aplicáveis à prática são aqueles que foram identificados pelo pesquisador ou pelo enfermeiro, independentemente de serem registrados no prontuário. Neste momento da produção de conhecimento sobre análise de aplicabilidade clínica não se considera pertinente estabelecer frequência mínima para que o elemento seja considerado aplicável, mas será um caminho necessário para que se possa produzir pesquisas com maior generalidade de resultados.

Para compor a análise, volta-se ao modelo teórico que sustentou o subconjunto e sugere-se que sejam avaliadas as contradições entre o modelo, os princípios e diretrizes do Sistema Único de Saúde e o modelo assistencial que é hegemônico no cenário de estudo. Após a análise é possível a adequação e atualização do subconjunto para que, de fato, represente o fenômeno, a clientela ou a especialidade a que se refere.

Por fim, discute-se que o modelo de terminologia de referência da norma ISO 18.104 representa estruturas de modelo clínico detalhado com grande potencial para contribuir na análise de aplicabilidade. Esta situação foi observada na pesquisa que identificou atributos dos eixos foco e julgamento e os domínios físico e psicológico para avaliação de enfermagem de cuidados perinatais^(30)^, cujo modelo foi usado para categorizar atributos e domínios. Como estudo futuro, o método aqui descrito deverá valer-se do conteúdo da ISO 18.104 para melhorar a coleta de dados e a análise dos resultados.

Um quadro-resumo do percurso metodológico para avaliação de aplicabilidade clínica de subconjunto terminológico na APS está representado na Figura 1.

**Figura 1:** Quadro-resumo das etapas do método de análise de aplicabilidade clínica de subconjunto terminológico na APS. Curitiba, 2025.

## LIMITAÇÕES DO MÉTODO

Como método inovador e pouco explorado, ele possui limitações. Nos pré-requisitos, pode haver dificuldades para: a) selecionar enfermeiro com habilidade clínica; b) operacionalizar espaços de educação permanente com o tema da CIPE® e modelos teóricos. No que se refere à observação das consultas, uma limitação a ser considerada é o fato de que o pesquisador também deve ter experiência com consulta de enfermagem. Estes cenários são de difícil convergência. Ademais, o método carece de um debate relacionado ao número de consultas observadas a depender da prevalência do fenômeno ou da quantidade de consultas de enfermagem realizadas no contexto analisado. Outra limitação refere-se à ausência de critérios estatísticos para o dimensionamento da amostra. Acredita-se que, com o uso progressivo do método, as limitações serão superadas. Por fim, a incipiência de estudos internacionais sobre o tema limita a comparação entre países.

## CONSIDERAÇÕES FINAIS

O método descrito é composto de três pré-requisitos e quatro etapas. Ele poderá ser replicado em cenários da APS de modo a ser criticado, ajustado e reorganizado. Acredita-se que subconjuntos submetidos a avaliação de aplicabilidade clínica têm potencial para oferecer acurácia aos elementos que o compõe e para representar as necessidades das pessoas, famílias ou comunidades a quem se destinam os subconjuntos. Considera-se que a implantação de um sistema classificatório para representar as práticas da enfermagem na APS não é garantia de que a equipe de enfermagem estabeleça um processo de cuidado ou de trabalho aderente à visão de mundo da saúde coletiva, tampouco ao modelo teórico que sustenta o subconjunto. Para isso, são necessárias incorporações de mudanças do modelo hegemônico e discussões/apropriações sobre os pressupostos teóricos conceituais, tanto do saber específico da enfermagem derivado de teorias próprias, quanto do domínio da saúde coletiva. Entretanto, compreende-se que o método é um instrumento para analisar a práxis.

## Data Availability

Todo o conjunto de dados que dá suporte aos resultados deste estudo foi publicado no próprio artigo. **Apoio Financeiro:** Conselho Nacional de Desenvolvimento Científico (CNPq) - Processos: 403654/2021-1 (Chamada Universal CNPq/MCTI/FNDCT N. 18/2021 - Faixa B - Grupos Consolidados) e 307054/2021-7 (Chamada CNPq Nº 4/2021 - Bolsas de Produtividade em Pesquisa – PQ).

# Translation: Método de análisis de la aplicabilidad clínica de un subconjunto terminológico en la atención primaria de salud: estudio teórico^*^

**DOI:** 10.1590/1980-220X-REEUSP-2025-0098es

**Authors:** Marcia Regina Cubas, Francine Dutra Mattei, Fernanda Broering Gomes Torres, Denilsen Carvalho Gomes

**Affiliations:** 1 Pontifícia Universidade Católica do Paraná, Programa de Pós-graduação em Tecnologia em Saúde, Curitiba, PR, Brasil.; 2 Pontifícia Universidade Católica do Paraná, Curso de Enfermagem, Curitiba, PR, Brasil.; 3 Secretaria Municipal da Saúde de São José dos Pinhais, Escola de Saúde Pública, São José dos Pinhais, PR, Brasil.

**Keywords:** Terminología Normalizada de Enfermería, Métodos, Enfermería de Atención Primaria

## Abstract

**Objetivo:**

Detallar las etapas del método para el análisis de la aplicabilidad clínica de subconjuntos terminológicos en el contexto de la Atención Primaria de Salud.

**Métodos:**

Estudio teórico-reflexivo que presenta tres requisitos previos y cuatro etapas del método para el análisis de la aplicabilidad clínica de subconjuntos terminológicos, a partir de la reflexión sobre dos experiencias de operacionalización.

**Resultados:**

Los requisitos previos están relacionados con la elección del escenario de la investigación, la selección de los participantes (clientes y enfermeros), con énfasis en los criterios de inclusión, y el conocimiento previo de la terminología y el modelo teórico. Las etapas del método son: elaboración del instrumento de recolección de datos, de acuerdo con las etapas del Proceso de Enfermería y las categorías del modelo teórico; observación, sistemática y participante, de la consulta de enfermería; evaluación de la consulta de retorno en la unidad de salud o en el domicilio; organización y análisis de los datos, de manera descriptiva y de acuerdo con el modelo teórico. De manera complementaria, se puede elaborar un estudio de caso ejemplar.

**Consideraciones finales:**

El método presentado puede replicarse en los escenarios de la Atención Primaria de Salud, con el fin de ser criticado, ajustado y reorganizado. Se entiende que el método es un instrumento para analizar la praxis.

## INTRODUCCIÓN

Desde 2008, el Consejo Internacional de Enfermería (*International Council of Nurses* - ICN) propone que el uso de la Clasificación Internacional para la Práctica de Enfermería (CIPE®) se facilite mediante subconjuntos terminológicos dirigidos a una determinada clientela o especialidad o foco de atención de la enfermería. La propuesta se justifica por la complejidad y extensión de la CIPE®, que dificulta su uso en la práctica. A este problema se suma la incipientes familiaridad de los enfermeros con los sistemas de clasificación representados por modelos ontológicos jerárquicos. En el ámbito de la enfermería, los modelos ontológicos jerárquicos permiten la combinación de conceptos primitivos para la composición de conceptos precoordinados. En el caso de la CIPE®, los diagnósticos, los resultados y las intervenciones de enfermería se consideran conceptos precoordinados.

Para la elaboración de subconjuntos, el CIE presentó en 2008 una guía compuesta por 10 pasos, posteriormente se publicó un modelo de seis pasos relacionados con el ciclo de vida de la terminología y, en 2015, se dio a conocer el método brasileño para el desarrollo de subconjuntos con tres requisitos previos y cuatro etapas organizativas^(1)^. Los subconjuntos terminológicos son relevantes para la representación de los fenómenos de enfermería en terminologías de referencia, entre las cuales se encuentra la *Systematized Nomenclature of Medicine - Clinical Terms* (SNOMED-CT). La mayoría de los conceptos precoordinados de la CIPE® 2019, que está representada por la SNOMED-CT, tienen su origen en los subconjuntos aprobados por el ICN. Un estudio que comparó las representaciones de los cuidados de enfermería a personas con COVID-19 en la CIPE® 2019, en la SNOMED-CT y en la correspondencia entre la CIPE® y la SNOMED-CT, concluyó que la SNOMED-CT abarca mejor la situación clínica del paciente^(2)^. Esta conclusión motiva el debate de que la CIPE® mejorará la representación de sus conceptos precoordinados al beneficiarse de los términos de SNOMED-CT. Para que la mejora sea efectiva, se necesitan investigaciones que analicen la aplicabilidad de los subconjuntos, a fin de identificar el uso de términos en la práctica asistencial, incluidos los que no figuran en la CIPE®, pero sí en SNOMED-CT.

La propuesta del método brasileño para el desarrollo de subconjuntos terminológicos surgió de un debate sobre la problemática de la falta de estandarización y detalle del recorrido metodológico en las investigaciones sobre el tema^(1)^. La motivación de las autoras se centró en proporcionar un método reproducible, cuyos resultados generados fueran comparables y reutilizables en otros contextos nacionales e internacionales.

Así, con la presencia de la guía del ICN, considerada como el método oficial, se observaron dos situaciones en las investigaciones que utilizaron la guía. La primera, que algunas de sus etapas se modificaban, excluían o incluían. La segunda, que los motivos o justificaciones para tales cambios, exclusiones o inclusiones no se explicaban. Tales situaciones desacreditaban científicamente las investigaciones que desarrollaban subconjuntos y dificultaban la reproducibilidad del método^(3)^.

Los investigadores brasileños protagonizan la producción de investigaciones sobre subconjuntos terminológicos en varios dominios del conocimiento y áreas temáticas de la Enfermería. Una revisión de la literatura realizada entre los años 2008 y 2017 identificó 35 producciones sobre el tema, de las cuales 31 estaban vinculadas a programas de posgrado brasileños^(3)^. En el artículo se indican las potencialidades de las producciones, entre ellas, la presencia de un modelo o referencial teórico que sustentó el subconjunto. Sin embargo, se describen algunas lagunas, entre ellas la falta de especificación o detalle de la etapa de validación de los conceptos por parte de especialistas y la incipiencia de los estudios de validación clínica^(3)^. Así, se plantea la cuestión sobre la finalidad principal de un subconjunto terminológico: su aplicación en la práctica clínica; por lo tanto, los estudios de validación clínica se consideran esenciales para la consolidación de la terminología^(4)^.

Una revisión del alcance de la validación clínica de los subconjuntos de la CIPE®, publicada en 2025, seleccionó 15 estudios, entre artículos, tesis y disertaciones^(5)^. Los principales resultados descritos por los autores son: que Brasil es el mayor productor de investigaciones sobre el tema, siendo responsable de 14 de los 15 estudios; 11 estudios se desarrollaron en el contexto hospitalario y 10 utilizaron el estudio de caso como método^(5)^. En el contexto de la APS, se identificó un estudio de precisión de indicadores clínicos, en el fenómeno de las enfermedades crónicas, y otro estudio, dirigido a las personas mayores, en el que se incluyó la observación directa de la consulta de enfermería como etapa del método^(5)^. A pesar de la aplicación cuidadosa de los métodos utilizados en las investigaciones seleccionadas, los autores concluyen que es necesario uniformizar el método^(5)^, siendo esta laguna de conocimiento la que se pretende subsanar con el presente artículo.

Cabe destacar que, en el entorno hospitalario, las etapas del proceso de enfermería se desarrollan de forma continua durante el periodo de hospitalización, incluyendo a diferentes enfermeros y equipos. En la Atención Primaria de Salud (APS), el Proceso de Enfermería se pone en práctica en el momento de la consulta de enfermería, realizada por el enfermero, que involucra al equipo de salud en las etapas que le corresponden legalmente. Las características de las prácticas en los distintos contextos determinan que el método de análisis de aplicabilidad en el hospital sea distinto al de la APS. De este modo, se justifica el detalle diferenciado del método.

Tras la publicación del método brasileño para el desarrollo de subconjuntos terminológicos, en 2015, a través de un capítulo de un libro y, en 2017, mediante un artículo que comparaba los tres métodos disponibles^(1)^, ha sido utilizado por varios investigadores. En el ámbito de la APS se pueden citar ejemplos de subconjuntos: para personas con tuberculosis^(6)^y para personas alcohólicas^(7)^. En el ámbito hospitalario, algunos ejemplos son los subconjuntos: para personas con enfermedad renal crónica en tratamiento conservador^(8)^ y para personas con quemaduras^(9)^. Aunque se han superado las cuestiones relacionadas con el detalle de las etapas para el desarrollo de subconjuntos, persiste la limitación de que la etapa de validación clínica no es obligatoria.

Diez años después de la publicación del método brasileño para el desarrollo de subconjuntos, este debe actualizarse como consecuencia de la migración de CIPE® a SNOMED-CT. En particular, en lo que se refiere al modelo ontológico jerárquico de los conceptos primitivos que sustenta el mapeo cruzado entre los términos identificados y los términos de la clasificación de referencia. Hasta el momento, no existe una posición del ICN sobre cómo se desarrollarán los subconjuntos, lo que no interfiere en el hecho de que es necesario estructurar el análisis de aplicabilidad de los subconjuntos ya elaborados en el contexto brasileño.

La elaboración de un método para el análisis de la aplicabilidad clínica se inició mediante asociaciones con los desarrolladores del subconjunto para morir con dignidad y del subconjunto de lactancia materna. Aunque los acercamientos fueron importantes y esclarecedores, las dos experiencias no se llevaron a cabo en espacios de APS y utilizaron el término validación, sin acercarse a la propuesta de análisis de aplicabilidad clínica en el contexto de la APS. La primera validó el subconjunto para pacientes en cuidados paliativos en hospitales y hospicios^(10)^. La segunda validó las intervenciones en puérperas en el posparto inmediato y al final del período, mediante llamada telefónica^(11)^.

Se corrobora la afirmación de que la inexistencia de validación clínica impide que el subconjunto pueda reflejar exactamente lo que se propone^(3)^. A su vez, las investigaciones que realizaron la validación clínica de subconjuntos reforzaron dos limitaciones relevantes: a) los métodos tradicionales de validación clínica se dirigen, prioritariamente, a uno de los elementos del Proceso de Enfermería: el diagnóstico^(12,13)^ y, a partir de la década de 2020, al resultado de enfermería^(14)^; b) las investigaciones se basan en un paradigma difícil de reproducir cuando se trata de un conjunto de diagnóstico, resultado e intervención de enfermería, en concreto en lo que se refiere al número de participantes y a la muestra. Esta última limitación no es exclusiva de la CIPE®. Se verificó en el estudio que evaluó la validez del contenido del diagnóstico de enfermería “Procesos familiares interrumpidos” de la NANDA-I en mujeres privadas de libertad, en el que los autores indican que el pequeño tamaño de la muestra puede generar un valor sesgado^(13)^. Por lo tanto, las investigaciones sobre la aplicabilidad clínica de subconjuntos deben considerar la cuestión de la muestra como una barrera y buscar alternativas para superarla.

A su vez, los métodos de validación están relacionados, en general, con las investigaciones que evalúan instrumentos de medición y se basan en el análisis psicométrico^(15)^. De este modo, se defiende la hipótesis de que, en las investigaciones con subconjuntos terminológicos, el término validación puede no alcanzar su alcance o, en el mejor de los casos, se utilizará de forma restringida. Entendiendo que el objetivo principal es analizar si el subconjunto tiene potencial para ser utilizado en la práctica clínica de los enfermeros, es decir, en la operacionalización de la consulta de enfermería en la APS, se defiende que el término más adecuado al contexto es aplicabilidad clínica.

El objetivo de este artículo es detallar las etapas del método para el análisis de la aplicabilidad clínica de los subconjuntos terminológicos, en el contexto de la Atención Primaria de Salud.

## MÉTODO

Estudio teórico-reflexivo, que presenta los requisitos previos y las etapas de un método para el análisis de la aplicabilidad clínica de subconjuntos terminológicos, en el contexto de la APS.

Se utilizó una reflexión de 2015 sobre la validación de los subconjuntos terminológicos de la CIPE®^(16)^ para la composición inicial de las etapas del método de aplicabilidad clínica en la APS. Las autoras de la reflexión debatieron modelos de validación y sugirieron, de forma preliminar y sin entrar en detalles, cuatro etapas que se pondrían en práctica mediante estudios de casos clínicos. De las etapas sugeridas, el método descrito en el presente artículo utilizó la elaboración del instrumento de recolección de datos de acuerdo con el modelo teórico, con posterior aplicación de una prueba piloto; y el análisis de los diagnósticos, resultados e intervenciones identificados en la práctica^(16)^.

El método de aplicabilidad clínica de subconjuntos terminológicos en la APS es parte integrante del texto reflexivo defendido por la primera autora en el concurso de Libre Docente en el Departamento de Enfermería en Salud Colectiva de la Escuela de Enfermería de la Universidad de São Paulo (EEUSP), en 2023. Su elaboración se derivó de la inclusión del método en un proyecto de investigación promovido por la Convocatoria Pública del Edicto Universal del Consejo Nacional de Desarrollo Tecnológico/Ministerio de Ciencia y Tecnología/ Fondo Nacional de Desarrollo Científico y Tecnológico (CNPq/ MCTI/FNDCT) n.º 18/2021.

La investigación derivada de la convocatoria se llevó a cabo en los años 2023 y 2024, en consultas de enfermería en la APS de un municipio de la región metropolitana de Curitiba (Paraná), para adultos con dolor crónico. Además, el método se utilizó para analizar la aplicabilidad de un subconjunto de desarrollo infantil, con niños de cero a tres años de edad, en el mismo municipio. Para componer el presente artículo, se utilizaron los textos descriptivos del método en los proyectos de origen, cuyas etapas fueron detalladas, justificadas y adaptadas tras su aplicación en los fenómenos del dolor crónico y el desarrollo infantil.

Las investigaciones que utilizaron el método fueron aprobadas por el Comité de Ética en Investigación (CEP) de la Pontificia Universidad Católica de Paraná, bajo el número CAAE 56765322.1.0000.0020, el 16 de abril de 2022, y CAAE 70495723.0.0000.0020, el 11 de agosto de 2023, y por el CEP de la Secretaría de Salud de São José dos Pinhais, bajo CAAE 56765322.1.3002.9587, el 2 de mayo de 2023, y CAAE 70495723.0.3001.9587, el 28 de agosto de 2023.

## REQUISITOS PREVIOS

Inicialmente, se observó que algunos puntos preceden a las etapas, siendo considerados requisitos indispensables para el análisis de la aplicabilidad del subconjunto terminológico en la APS. En este sentido, se incluyen tres requisitos previos: a) elección del escenario de estudio; b) selección de los participantes en el estudio; c) conocimiento de la terminología, el modelo teórico y las definiciones operativas de los diagnósticos y resultados de enfermería del subconjunto terminológico.

Es necesario detallar y justificar los requisitos previos. En el primero, la elección del escenario, el fenómeno al que se refiere el subconjunto debe ser prevalente o la clientela debe ser usuaria habitual del lugar de estudio. No hay duda de que, si el fenómeno no es prevalente, habrá un obstáculo para la selección de participantes y la identificación de los diagnósticos, resultados e intervenciones puede verse perjudicada por la falta de atributos definidores. Se añade la justificación de que, en investigaciones sobre la validación del contenido de los enunciados del subconjunto, mediante la técnica Delphi, se ha comprobado que cuando los fenómenos pertenecen al ámbito de competencia de la enfermería, tiende a haber un debate con mayor contribución a la eliminación o adición de elementos^(8,17)^. Por lo tanto, se defiende la hipótesis de que si el enfermero asistencial reconoce el fenómeno en su rutina, será capaz de identificar con mayor claridad los elementos del proceso de enfermería susceptibles de ser representados por terminología estandarizada.

En relación con el segundo requisito previo, la selección de los participantes, es importante considerar criterios adecuados para los enfermeros y los usuarios. Los enfermeros que serán seleccionados o invitados a participar en el estudio deben tener obligatoriamente experiencia clínica, es decir, la consulta de enfermería dirigida al fenómeno o la clientela debe ser una rutina asistencial diaria o semanal. Se sugiere un criterio de inclusión relacionado con el tiempo como enfermero asistencial de al menos dos años. Esta temporalidad se utilizó en las investigaciones que aplicaron el método aquí descrito. También se utilizó en la investigación que analizó los diagnósticos de enfermería en la asistencia domiciliaria^(18)^.

La habilidad clínica desarrollada por el ejercicio de la consulta de enfermería es un tema inmerso en contradicciones. El tiempo de formación y trabajo en la APS no siempre garantiza la habilidad clínica. Este hecho se observó en un estudio que analizó la práctica asistencial de 463 enfermeros de Paraíba, en el que los resultados describen que la consulta de enfermería fue reportada como práctica principal por el 79,7% de los enfermeros^(19)^. Sin embargo, cuando se analizó la percepción de la resolución, el resultado fue del 65%, y el tiempo de formación y trabajo en la APS varió de uno a 20 años^(19)^. Por lo tanto, se sugiere que el criterio de inclusión de los enfermeros participantes no se dirija únicamente al tiempo de formación o al tiempo de trabajo en la APS, sino al ejercicio efectivo de la consulta de enfermería a los clientes que experimentan el fenómeno investigado, incluidas las consultas realizadas en el domicilio. También se recomienda encarecidamente que el investigador tenga experiencia en la operacionalización de la consulta de enfermería en la APS, ya que, de lo contrario, puede representar un sesgo en el estudio, por la falta de identificación con el proceso de enfermería.

Otra sugerencia que debe evaluarse al describir los criterios de selección o inclusión de los enfermeros es la formación académica *lato* o *stricto sensu*. Se defiende que, para la aplicabilidad clínica, tener un posgrado, aunque es deseable, no es el criterio central o principal para la elección. En la propuesta de instrumentos para la selección de especialistas para el desarrollo de subconjuntos terminológicos, se establecieron 13 criterios en la etapa de aplicabilidad clínica distribuidos en cinco dominios clasificadores, siendo los dominios de la experiencia clínica y el tiempo de práctica profesional los atribuidos como relevantes^(20)^.

A su vez, se identifica en la literatura que la residencia multi-profesional en salud familiar tiene el potencial de transformar las prácticas en los servicios, a pesar de las limitaciones relacionadas con las debilidades de los servicios y las trayectorias formativas^(21)^. Cabe añadir que tener una residencia en salud fue uno de los criterios de inclusión de especialistas en un estudio que identificó diagnósticos de enfermería de la CIPE® en personas atendidas en un programa de asistencia domiciliaria^(18)^. De este modo, si en el escenario del estudio existe la posibilidad de seleccionar enfermeros con formación en residencia en salud familiar, el criterio puede ser un calificador para el análisis de la aplicabilidad clínica, por la característica de la formación.

En cuanto a los usuarios participantes, atendidos por la consulta de enfermería, es necesario determinar con parsimonia los criterios de inclusión para representar adecuadamente el fenómeno abordado. Así, uno de los criterios sugeridos es la capacidad cognitiva del usuario para comprender información sencilla, ya que puede ser un elemento de confusión para la identificación de atributos subjetivos o para la comprensión de las intervenciones de enfermería.

Si no es posible incluir el criterio, se debe realizar un análisis del sesgo de confusión, indicándolo como límite de la investigación. Una alternativa para minimizar el sesgo es separar los resultados según las características cognitivas de los participantes. En relación con el tercer requisito previo, el conocimiento de la terminología, el modelo teórico y las definiciones operativas, se sugiere una fase previa a la recolección de datos. En ella, se presentarán la CIPE®, el conjunto de definiciones operativas y el modelo teórico. La forma en que se llevará a cabo este momento es a elección del investigador. Como ejemplo, en el análisis del subconjunto de personas con dolor crónico, las enfermeras participaron en tres talleres con contenido preparatorio, de ocho horas de duración cada uno, y fueron participantes de la investigación que validó las definiciones operativas^(22)^. Este requisito previo se justifica por el hecho de que la CIPE® no es el sistema de clasificación hegemónico en Brasil, por lo que el análisis de la aplicabilidad clínica puede verse perjudicado por la falta de comprensión de los componentes de la clasificación.

Sin embargo, se discute que los términos primitivos de la CIPE® representan situaciones cotidianas de la asistencia, como los resultados presentados en el artículo que elaboró el subconjunto para personas con úlcera del pie diabético en la APS^(23)^. En el ámbito internacional, una investigación realizada en Polonia, que exploró el uso de la CIPE® como parte del desarrollo y el aprendizaje profesional, en la que participaron 772 enfermeros, presenta en su conclusión que, a pesar de las dificultades para implementar la clasificación en la práctica clínica, los procesos educativos ayudan al desarrollo de habilidades y a la comprensión del uso de la CIPE® en el proceso de enfermería^(24)^. Se defiende que el momento previo a la presentación también se configura como una estrategia de educación permanente en el servicio y que la familiarización de los enfermeros con terminologías estandarizadas puede mejorar el razonamiento clínico, ayudar a registrar el fenómeno de forma organizada y permitir la recuperación de información estructurada.

En cuanto al conocimiento del modelo teórico, es importante recordar que los subconjuntos terminológicos se organizan según los conceptos de una teoría o modelo, incluidas las Teorías de Medio Alcance (TMA) elaboradas específicamente para ellos, como la Teoría Interactiva de la Lactancia^(25)^ y la Teoría del Cuidado en el Contexto del Riesgo Cardiovascular^(26)^. En el contexto de la APS, una revisión de alcance que mapeó las evidencias sobre el uso de referencias teóricas por parte de los enfermeros identificó 32 teorías o modelos empleados en la recolección de datos, en la elaboración de TMA o para subsidiar la atención^(27)^. La teoría más utilizada fue la teoría del déficit de autocuidado de Orem, seguida por las teorías de adaptación de Roy, de alcance de metas de King y la teoría de Parse^(27)^.En la revisión se discute que los enfermeros de la APS aplican los supuestos de las grandes teorías y, de forma discreta, las TMA, destacando que estas últimas pueden atender las demandas de cuidado por su capacidad de operar en el nivel abstracto de las grandes teorías^(27)^. Se considera que la aproximación de los enfermeros de APS a las grandes teorías, las TMA o los modelos teóricos utilizados en el subconjunto terminológico potencia la comprensión del alcance de su aplicabilidad clínica y sustenta la traducción del conocimiento.

Por último, se retoma la afirmación sobre el criterio considerado de mayor relevancia para la elección del enfermero participante: la habilidad clínica. La experiencia del enfermero con las investigaciones relacionadas con el CIPE®, las teorías o el Proceso de Enfermería es importante para el desarrollo de un subconjunto. Sin embargo, el análisis de su aplicación en la práctica está determinado por la capacidad que tiene el enfermero asistencial para reconocer un fenómeno, planificar y ejecutar intervenciones y analizar los resultados esperados o alcanzados. Si en el proceso de selección se identifica a un enfermero con experiencia en investigación con CIPE® y/o el Proceso de Enfermería, pero con poca experiencia clínica en APS, corresponde al investigador analizar la pertinencia de su participación y dejar clara la justificación para incluirlo.

## ETAPAS

### Etapa 1 - Elaboración del Instrumento de Recolección de Datos

La forma de recolección de datos para el análisis de la aplicabilidad clínica es la observación sistemática y participante de la consulta de enfermería. El instrumento de recolección de datos debe construirse de acuerdo con las etapas del Proceso de Enfermería y organizarse de acuerdo con el modelo teórico que sustentó el subconjunto. Debe contener: a) elementos para la evaluación inicial (datos sociodemográficos, anamnesis, datos antropométricos y examen físico), cuyos datos tienen el potencial de subsidiar la identificación de diagnósticos, resultados e intervenciones de enfermería; b) lista de diagnósticos, resultados e intervenciones de enfermería del subconjunto; c) elementos para la evolución de la enfermería. Al tratarse de un instrumento no estandarizado y elaborado de acuerdo con el alcance del subconjunto terminológico, es necesario realizar una prueba piloto para verificar la comprensión de las preguntas y el contenido técnico y para adaptarlo a la rutina de la consulta de enfermería en el escenario de recolección de datos. La prueba piloto puede aplicarse a enfermeros asistenciales con características similares a los que serán seleccionados para la investigación, con el fin de verificar la claridad y pertinencia de los elementos presentes en el instrumento.

El instrumento de recolección de datos debe ser completado por la investigadora, pero, en caso de que se involucren otros recopiladores, será necesario capacitarlos para el uso y la orientación de los procedimientos de observación sistemática. Es importante afirmar que no se trata de una evaluación sobre la forma en que se opera la consulta de enfermería, sino si el subconjunto terminológico es aplicable en la práctica asistencial. Por lo tanto, no deben incluirse cuestiones relacionadas con la forma de la consulta (por ejemplo, si el enfermero realizó el examen físico correctamente) o elementos de recursos (porejemplo, si hay suficientes recursos humanos o materiales), ni tampoco cuestiones relacionadas con el modelo de gestión (por ejemplo, si la dirección de enfermería apoya la implantación de terminologías estandarizadas).

Se sugiere que, si es necesario contextualizar la operacionalización de la consulta de enfermería por su relevancia en el escenario de la investigación, la información debe figurar en la descripción del lugar de estudio. A riesgo de repetirnos, antes de la recolección de datos, los enfermeros deben tener acceso al subconjunto terminológico, estructurado de acuerdo con el modelo teórico.

Es necesario aclarar las dudas, especialmente las relacionadas con la clasificación, ya que no se trata de una terminología hegemónica en la práctica y la enseñanza de la enfermería brasileña. Aunque existen experiencias puntuales, como es el caso de la inclusión de la CIPE® en la APS, junto con la Clasificación Internacional de Atención Primaria (CIAP®), en los protocolos del estado de Santa Catarina^(28)^. Se cree que el desconocimiento de la CIPE®, así como la lejanía del contenido del subconjunto y de la teoría básica, pueden considerarse sesgos de selección de los participantes en el estudio. Es decir, el subconjunto terminológico puede no ser aplicable porque los enfermeros desconocen los supuestos de la teoría que lo sustentó y desconocen su contenido.

### ETAPA 2 - Observación de la Consulta de Enfermería

Antes de describir esta etapa, se discute la problemática del número de consultas que se observarán. En una reflexión anterior sobre el tema no se sugiere una cantidad^(16)^. En las dos experiencias de análisis de aplicabilidad en espacios hospitalarios, el número fue de 20 personas^(10,11)^. Lo que cambió en las investigaciones fue el número de observaciones por persona, dadas las características de la población del estudio, el fenómeno abordado y el resultado esperado.

En el espacio ambulatorio hospitalario, se identificó una experiencia de validación clínica de un subconjunto terminológico para personas con enfermedad renal crónica en tratamiento conservador^(8)^. En la investigación se incluyeron, de antemano, 50 pacientes hospitalizados y evaluados al ingreso, a los 30 días y a los 60 días de hospitalización, cuyos datos se utilizaron en la elaboración de estudios de casos múltiples con posterior análisis por parte de enfermeros especialistas en relación con la concordancia con los diagnósticos, resultados e intervenciones de enfermería elaborados por los investigadores^(8)^. Aunque se trata de una excelente aproximación al tema, debido a que los estudios de casos se construyen con datos reales, persiste la limitación de la inclusión de enfermeros especialistas para analizar el subconjunto terminológico. Además, no se analiza la aplicabilidad clínica en las consultas realizadas por enfermeros asistenciales en el ambulatorio, lo que constituye la laguna contemplada en el presente estudio teórico.

Para determinar el número de participantes en el análisis de aplicabilidad clínica del subconjunto terminológico en la APS, se sugiere considerar dos cuestiones: el número de consultas ofrecidas mensualmente por el enfermero del escenario de estudio y la previsión de atención a la clientela que experimenta el fenómeno, en el mismo período. A partir de ahí, en un primer momento, se puede optar por observar todas las consultas realizadas en el mes o durante un periodo de tiempo más largo hasta alcanzar, como mínimo, las 20 consultas. En las dos experiencias de puesta en práctica del método no se estableció un cálculo de la muestra y la recolección de las 20 consultas, iniciales y de seguimiento, tuvo una duración media de tres meses, con visitas semanales a la unidad de salud.

La consulta de enfermería será realizada por el enfermero de la unidad de salud y observada por el investigador, sin interferencia. El usuario de la consulta de enfermería debe ser informado sobre el papel del investigador en este proceso y sobre la investigación, ya que también es participante de la misma. De este modo, se ofrecerá el formulario de consentimiento libre e informado al enfermero y a la persona consultada, respetando los requisitos éticos de la investigación que involucra a seres humanos.

Los diagnósticos, los resultados esperados y las intervenciones de enfermería serán identificados por el investigador y el enfermero, de forma individual y sin intercambio de información. El enfermero lo registrará en el expediente médico, según la rutina de la unidad de salud. Es importante destacar que el hecho de que el servicio no disponga de un espacio específico en el expediente médico para registrar los elementos que componen el subconjunto terminológico no impide que se realice la investigación, ya que se espera que el registro de las etapas del proceso de enfermería se realice de acuerdo con la legislación específica de la profesión.

Una vez finalizada la consulta, el investigador y el enfermero compararán los diagnósticos, los resultados esperados y las intervenciones seleccionadas e indicarán la similitud, concordancia o discordancia entre los elementos (diagnósticos, resultados e intervenciones) observados por el investigador, identificados por el enfermero y registrados por el enfermero.

Además de identificar similitudes, coincidencias o discrepancias, la comparación tiene como objetivo verificar la observación realizada y los registros realizados. Se deben agrupar para su posterior análisis: a) elementos observados por el investigador y no identificados por el enfermero; b) elementos identificados por el enfermero y no observados por el investigador; c) elementos identificados por el enfermero y no registrados; d) elementos registrados por el enfermero y no observados por el investigador; e) elementos observados por el investigador, identificados y registrados por el enfermero.

### ETAPA 3 - Evaluación en la Consulta de Seguimiento

Al final de la primera consulta se programarán una o más consultas de seguimiento para la evaluación posterior, a fin de cumplir con la etapa de evolución de enfermería del Proceso de Enfermería. El plazo sugerido es de hasta 30 días, dependiendo del fenómeno, la clientela o el modelo teórico. La necesidad de más de una consulta de seguimiento depende del diagnóstico de enfermería identificado y de la temporalidad de la intervención propuesta.

La consulta de seguimiento puede realizarse en la unidad de salud o en el domicilio y debe ser realizada, prioritariamente, por el enfermero que realizó la consulta inicial. Si no es posible que él realice la consulta, esta podrá ser realizada por el investigador que recopiló los datos. En la consulta de seguimiento se identificarán: el resultado alcanzado o sensible a la intervención propuesta, la adherencia a las intervenciones y la identificación, si procede, de un nuevo diagnóstico.

Cabe destacar que la consulta de enfermería de seguimiento orienta la práctica profesional, permitiendo el seguimiento sistemático de los fenómenos y condiciones de salud y posibilitando la evaluación de la adherencia de la persona/familia a las intervenciones propuestas. Este tema ha sido abordado en investigaciones recientes, como el estudio que identificó el autogestión de la práctica de ejercicios y la adherencia a la medicación en pacientes de riesgo en la APS^(29)^, lo que permitió la ejecución de todas las etapas del proceso de enfermería en su totalidad.

### ETAPA 4 - Organización y Análisis de los Datos

Se sugiere que, después de cada consulta, los datos se introduzcan inmediatamente en una hoja de cálculo y sean verificados de forma independiente por otro investigador. Los datos recopilados deben organizarse o categorizarse según el modelo teórico y describirse en frecuencia absoluta y relativa de los elementos de la práctica de enfermería.

Cabe destacar que los datos relacionados con las intervenciones de enfermería, debido a la cantidad de enunciados, pueden presentarse en conjunto, pero siempre respetando el modelo teórico y el diagnóstico de enfermería al que se refiere. Es importante analizar qué intervención se relacionó con el resultado de enfermería identificado en la consulta posterior, lo que servirá de apoyo para analizar los resultados sensibles a la acción de enfermería.

Además, los datos identificados en la evaluación inicial y en la consulta de seguimiento se utilizarán para componer estudios de casos ejemplares con la descripción de todas las etapas del proceso de enfermería relacionadas con las dos consultas de enfermería. La elaboración de estudios de casos ejemplares ayudará a analizar la adecuación de los diagnósticos, resultados e intervenciones que se consideraron aplicables y permitirá su uso en momentos de educación permanente en salud.

Se considera que los elementos aplicables a la práctica son aquellos que han sido identificados por el investigador o por el enfermero, independientemente de que estén registrados en el expediente médico. En este momento de la producción de conocimientos sobre el análisis de la aplicabilidad clínica, no se considera pertinente establecer una frecuencia mínima para que el elemento se considere aplicable, pero será un camino necesario para poder producir investigaciones con resultados más generales.

Para componer el análisis, se vuelve al modelo teórico que sustentó el subconjunto y se sugiere evaluar las contradicciones entre el modelo, los principios y directrices del Sistema Único de Salud y el modelo asistencial que es hegemónico en el escenario de estudio. Tras el análisis, es posible adaptar y actualizar el subconjunto para que, de hecho, represente el fenómeno, la clientela o la especialidad a la que se refiere.

Por último, se discute que el modelo de terminología de referencia de la norma ISO 18.104 representa estructuras de modelo clínico detallado con gran potencial para contribuir al análisis de aplicabilidad. Esta situación se observó en la investigación que identificó los atributos de los ejes de enfoque y juicio y los dominios físico y psicológico para la evaluación de la enfermería de cuidados perinatales^(30)^, cuyo modelo se utilizó para categorizar atributos y dominios. Como estudio futuro, el método aquí descrito deberá valerse del contenido de la norma ISO 18.104 para mejorar la recolección de datos y el análisis de los resultados.

En la Figura 1 se presenta un cuadro resumen del recorrido metodológico para la evaluación de la aplicabilidad clínica del subconjunto terminológico en la APS.

**Figura 1:** Cuadro resumen de las etapas del método de análisis de aplicabilidad clínica del subconjunto terminológico en la APS. Curitiba, 2025.

## LIMITACIONES DEL MÉTODO

Al ser un método innovador y poco explorado, presenta algunas limitaciones. En cuanto a los requisitos previos, pueden surgir dificultades para: a) seleccionar enfermeros con habilidades clínicas; b) poner en marcha espacios de formación continua con el tema de la CIPE® y modelos teóricos. En lo que respecta a la observación de las consultas, una limitación a tener en cuenta es el hecho de que el investigador también debe tener experiencia en consultas de enfermería. Estos escenarios son difíciles de converger. Además, el método carece de un debate relacionado con el número de consultas observadas en función de la prevalencia del fenómeno o de la cantidad de consultas de enfermería realizadas en el contexto analizado. Otra limitación se refiere a la ausencia de criterios estadísticos para el dimensionamiento de la muestra. Se cree que, con el uso progresivo del método, las limitaciones se superarán. Por último, la incipiencia de los estudios internacionales sobre el tema limita la comparación entre países.

## CONSIDERACIONES FINALES

El método descrito consta de tres requisitos previos y cuatro etapas. Puede replicarse en escenarios de APS para ser criticado, ajustado y reorganizado. Se cree que los subconjuntos sometidos a evaluación de aplicabilidad clínica tienen el potencial de ofrecer precisión a los elementos que los componen y de representar las necesidades de las personas, familias o comunidades a las que se destinan los subconjuntos. Se considera que la implementación de un sistema de clasificación para representar las prácticas de enfermería en la APS no garantiza que el equipo de enfermería establezca un proceso de atención o de trabajo acorde con la visión del mundo de la salud colectiva, ni con el modelo teórico que sustenta el subconjunto. Para ello, es necesario incorporar cambios en el modelo hegemónico y debates/apropiaciones sobre los supuestos teóricos conceptuales, tanto del conocimiento específico de la enfermería derivado de sus propias teorías, como del ámbito de la salud colectiva. Sin embargo, se entiende que el método es un instrumento para analizar la praxis.

## Data Availability

Todo el conjunto de datos que apova los resultados de este estudio fue publicado en el propio artículo.
